# Immunoproteasomes in Skeletal Muscle Pathologies: Emerging Roles, Conflicting Evidence, and Future Directions

**DOI:** 10.3390/cells14201586

**Published:** 2025-10-12

**Authors:** Alexander Kalinkovich, Gregory Livshits

**Affiliations:** 1 Department of Anatomy and Anthropology, Gray Faculty of Medical and Health Sciences, Tel-Aviv University, Tel Aviv 6927846, Israel; kalinkovich@tauex.tau.ac.il; 2 Department of Morphological Sciences, Adelson School of Medicine, Ariel University, Ariel 4077625, Israel

**Keywords:** immunoproteasomes, skeletal muscle, sarcopenia, chronic inflammation

## Abstract

**Highlights:**

**What are the main findings?**

- Immunoproteasomes (IMPs) act as a double-edged sword in muscle biology; they promote protein quality control and muscle differentiation under normal or early disease conditions, but they can drive muscle wasting when persistently activated.
- Excessive IMP activity fosters chronic inflammation and harmful crosstalk between immune and muscle cells, thereby worsening degeneration.

**What is the implication of the main findings?**

- Targeting IMPs with selective inhibitors holds therapeutic promise in skeletal muscle diseases and experimental models of muscle loss.
- However, in skeletal muscle disorders, inhibition may be a mixed blessing, potentially reducing damaging inflammation but at the cost of impairing muscle maintenance and repair.

**Abstract:**

Skeletal muscle pathologies, including sarcopenia, inflammatory myopathies, and various muscular dystrophies, are strongly influenced by chronic low-grade inflammation and impaired proteostasis. Immunoproteasomes (IMPs), inducible proteolytic complexes activated by pro-inflammatory cytokines, are emerging as regulators linking immune signaling to protein quality control. Evidence suggests that IMPs have paradoxical, context-dependent roles in skeletal muscle. On one hand, they can support proteostasis and muscle regeneration under stress; on the other, persistent activation may sustain cytokine production, antigen presentation, and maladaptive immune–muscle interactions, promoting chronic inflammation and muscle wasting. Selective IMP inhibitors, such as ONX 0914 and KZR-616, display potent anti-inflammatory effects in preclinical models of autoimmune myositis and muscle atrophy. Yet, their use in skeletal muscle pathologies is controversial; while inhibition may dampen harmful immune activation, it could also impair muscle repair and proteostasis. This review summarizes current findings, highlights key contradictions, and explores unresolved questions about the role of IMPs in skeletal muscle pathologies. We emphasize the need for a deeper understanding of IMP-mediated mechanisms in skeletal muscle pathology and strategies combining selective inhibitors to enhance therapeutic efficacy while minimizing adverse effects. IMPs thus represent both a promising and potentially risky therapeutic target, with outcomes highly dependent on disease context.

## 1. Introduction

Sarcopenia is an age-related condition characterized by the progressive loss of skeletal muscle mass, strength, and function, and it is associated with serious adverse health outcomes, including increased risk of falls and fractures, functional decline, elevated healthcare costs, and mortality [1,2,3]. Although the etiology of sarcopenia is multifactorial [4], chronic low-grade inflammation has emerged as a central factor in its pathogenesis. This persistent inflammatory state contributes to muscle catabolism, oxidative stress, and impaired regenerative capacity not only in sarcopenia [5,6,7,8,9] but also in various muscular dystrophies [10] and inflammatory myopathies, such as polymyositis and dermatomyositis [11].

Imbalanced muscle protein turnover is another hallmark of sarcopenia [12] and other skeletal muscle pathologies [13,14]. This dysregulation is primarily governed by the ubiquitin–proteasome system (UPS), a highly conserved proteolytic pathway responsible for degrading damaged, misfolded, or short-lived regulatory proteins. Within the UPS, polyubiquitinated proteins are recognized and degraded by the 26S proteasome into short peptides and amino acids, thereby maintaining cellular proteostasis and preventing proteotoxic stress [15,16,17,18].

A specialized form of the proteasome, the immunoproteasome (IMP), is primarily expressed in immune cells, such as dendritic cells, macrophages, T lymphocytes, and natural killer cells, but it can also be induced in non-immune cells under inflammatory conditions. In IMPs, the constitutive catalytic β subunits β1, β2, and β5 are replaced by the inducible subunits β1i (LMP2), β2i (MECL1), and β5i (LMP7), respectively. Assembly of IMP is triggered by pro-inflammatory cytokines, particularly interferon-γ (IFNγ) and tumor necrosis factor-α (TNFα), which promote the substitution of the standard catalytic subunits with their inducible counterparts. This modification enhances the degradation of oxidized proteins and improves the generation of peptides for presentation on major histocompatibility complex class I (MHC-I) molecules, thus contributing to immune surveillance [19,20,21]. Beyond their role in immunosurveillance, accumulating evidence implicates IMPs in broader immunomodulatory functions, including the regulation of cytokine production, macrophage polarization, NLRP3 inflammasome activity, and inflammatory tissue damage [22,23].

However, within skeletal muscle biology, IMPs appear to play a nuanced and context-dependent role. Evidence suggests that skeletal muscle does express IMPs; for example, muscle biopsies from older adults have shown upregulation of IMP subunits, particularly β5i (LMP7), which correlates with increased expression of pro-inflammatory cytokines, such as IL-6 and TNFα [24]. Functional studies further support a role for IMPs in muscle homeostasis. Specifically, knockdown or pharmacological inhibition of β1i (LMP2) impairs C2C12 myoblast differentiation, increases protein oxidation and apoptosis, and inhibits myotube formation, highlighting a critical role for IMPs in myogenesis [25]. In vivo, β5i (LMP7) has been shown to support skeletal muscle regeneration by promoting the macrophage phenotype switch from pro-inflammatory (M1) to anti-inflammatory (M2). Inhibition of β5i (LMP7) in mice impairs muscle regeneration and increases interstitial fibrosis, further suggesting a beneficial role of IMPs in muscle repair [26].

Conversely, in certain pathological contexts, IMP activity appears detrimental to skeletal muscle function. In a murine model of muscular dystrophy, IMP inhibition alleviated muscle weakness and atrophy, likely through a reduction in muscle inflammation [27]. Similarly, in idiopathic inflammatory myopathies (IIMs), such as polymyositis and dermatomyositis, disorders characterized by immune-mediated muscle damage, akin in part to sarcopenia, there is constitutive overexpression of IMP subunits β1i (LMP2), β2i (MECL1), and β5i (LMP7) in affected muscle tissue [28,29]. Notably, in a mouse model of polymyositis, selective IMP inhibition preserved grip strength and reduced leukocyte infiltration into muscle, indicating a potentially harmful role for IMPs in inflammatory myopathies [30].

Despite these findings, no direct studies to date have examined the regulation of IMPs in sarcopenic muscle or their mechanistic connection to chronic inflammation. It remains unresolved whether IMP upregulation in aging muscle represents a compensatory mechanism aimed at maintaining proteostasis and supporting regeneration or a maladaptive response that exacerbates chronic inflammation, paralleling the pathological features observed in IIMs. An additional open question is whether IMP inhibition, while beneficial in inflammatory myopathies, would impair muscle regeneration in the context of sarcopenia due to the IMP’s role in myogenesis, or, conversely, whether it might attenuate chronic inflammation and thus help preserve muscle function in skeletal muscle pathologies.

This review aims to dissect the complex, context-dependent roles of immunoproteasomes in skeletal muscle pathologies, including but not limited to sarcopenia. We analyze current evidence on their involvement in regulating proteostasis, immune signaling, and inflammation and critically assess the therapeutic promise and risks of selective IMP inhibitors. By identifying key contradictions and unresolved questions, we seek to guide future strategies that balance immune modulation with the preservation of muscle function.

## 2. Immunoproteasomes: A Brief Overview

### 2.1. Structure

The structure of proteasomes and their role in proteostasis have been extensively described in numerous review articles, both under physiological conditions and in the context of various diseases, particularly cancer and neurodegenerative disorders (e.g., [31,32,33]). Much less is known about IMPs, although emerging evidence suggests their involvement not only in virus-induced inflammation [22,34] but also in disorders associated with chronic inflammation, such as autoimmune diseases [35,36,37] and neuroinflammatory disorders [38]. In this section, we briefly discuss the basic terms relevant to this article.

**Figure 1:**
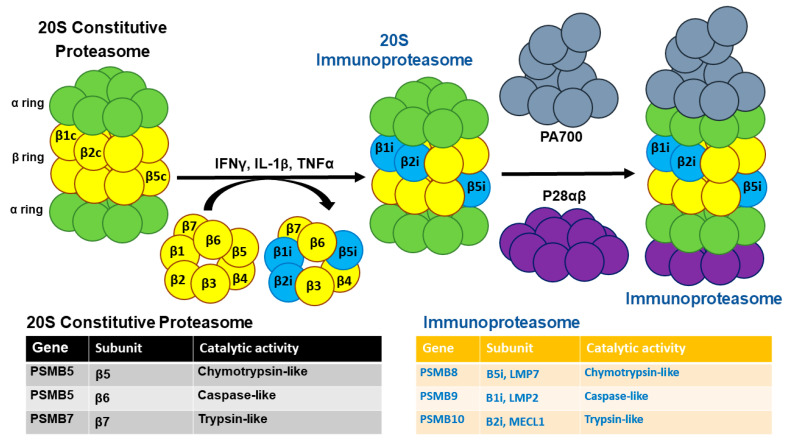
Simplified schematic of the immunoproteasome structure.

The proteasome is a critical component in the final step of protein degradation within cells, particularly within the UPS. It acts as a large, multi-subunit protease complex that degrades polyubiquitinated old, unused, and damaged proteins into peptides 8–21 amino acid long, which are further cleaved into amino acids for reuse by the ribosome [39]. As diagrammatically illustrated in Figure 1, the constitutive proteasome consists of a combination of two α and two β rings, each containing seven subunits. The β ring includes three standard catalytic subunits, β1c, β2c and β5c, each possessing caspase-like, trypsin-like, and chymotrypsin-like hydrolytic activity, respectively, which serve as the hydrolytic core of the proteasome [40,41]. In hematopoietic cells, including immune cells, and in cells stimulated with IFNγ, other pro-inflammatory molecules [42,43,44], or by oxidative stress [45], the catalytically active standard proteasome subunits β1c, β2c, and β5c in the inner two rings of the 20S proteasome are replaced by β1i (low molecular mass polypeptide 2 (LMP2, encoded by *PSMB9*), β2i (multicatalytic endopeptidase complex–like 1 (MECL-1), encoded by *PSMB10*), and β5i (LMP7, encoded by *PSMB8*), forming the IMP. In IMPs, proteasome activators PA700 (19S proteasome), PA28 α/β (11S proteasome), or a combination of both bind to 20S at its two ends. Notably, in IPMs, the caspase-like activity, exerted by β1c in standard proteasomes, is strongly reduced, whereas the chymotrypsin-like activity is enhanced [46,47,48]. This results in the generation of peptides with hydrophobic C-terminal residues, which are well-suited for presentation to MHC-I molecules [21].

As mentioned above, IMPs incorporate three inducible catalytic subunits, β1i (LMP2), β2i (MECL-1), and β5i (LMP7), which differ from their constitutive counterparts in both sequence (~60% identity) and substrate specificity, producing peptides better suited for MHC class I antigen presentation [49]. Importantly, IMPs do not form through subunit exchange in pre-existing 20S proteasomes. Instead, they assemble de novo following the induction of βi subunit expression. β1i is typically incorporated first, promoting the sequential addition of β2i and β5i [20,50]. This order contrasts with constitutive proteasome assembly, where β2 is usually incorporated first [51]. The selective incorporation of βi subunits is largely driven by their distinct propeptides, which influence chaperone interactions and subunit selection. Notably, IMP assembly is significantly faster, up to four times, than that of constitutive proteasomes, due in part to direct binding of β5i to the chaperone proteasome maturation protein (POMP) [38]. As a result of this coordinated assembly, most proteasomes in IFNγ-stimulated cells contain all three βi subunits, ensuring that IMPs dominate under inflammatory conditions [20,52,53].

Notably, there are currently no quantitative data available on the ratio between IMPs and constitutive 20S proteasomes in muscle fibers (or in other cell types) following exposure to pro-inflammatory stimuli or even under non-induced conditions. This gap in the literature makes it difficult to precisely assess how IMPs dynamically replace or coexist with standard proteasomes in these contexts [54]. This issue is further complicated by conflicting findings. Some studies suggest that IMPs more efficiently degrade polyubiquitinated proteins under IFNγ–induced inflammatory conditions [55], while others report no significant difference between standard and IMPs in this function [56]. These inconsistencies highlight the need for more precise, quantitative studies to clarify IMP dynamics and function in skeletal muscle.

The proteasomal core is composed of four stacked rings, two outer α rings and two inner β rings, each consisting of seven subunits. In response to pro-inflammatory stimuli, such as IFNγ, IL-1β, or TNFα, the β1, β2, and β5 catalytic subunits of the constitutive 20S proteasome are replaced by their immunoproteasome-specific counterparts, β1i, β2i, and β5i, forming the 20S immunoproteasome. This core particle can then associate at one or both ends with proteasome activators, such as PA700 (19S regulatory particle), PA28α/β (11S activator), or a combination of both, resulting in the formation of fully functional immunoproteasomes. A summary of the genes encoding these catalytic subunits and their associated enzymatic activities in both the constitutive and immunoproteasome forms is presented in the table at the bottom of the diagram.

### 2.2. Function

Recent findings indicate that proteasomes of most immune cell subsets mainly consist of IMP subunits [57]. Apart from MHC-I antigen processing, many processes have been attributed to the IMPs, including T cell expansion [47,58,59], the generation of Th17 cells while promoting regulatory T cell (Treg) development [60,61,62], macrophage polarization [63,64], protection from immunopathological damage in the brain [65,66], and transplant rejection [67]. IMPs are also considered to be involved in the development of a wide range of inflammation-associated diseases, including lung-related [68,69,70], neurodegenerative [71,72], cardiovascular (CVD) [73,74], and autoimmune diseases [35,75].

#### Inhibitors

The potential involvement of IMPs in the pathogenesis of various inflammation-associated disorders, as discussed above, positions IMPs as attractive therapeutic targets. To date, both nonselective and selective IMP inhibitors have been developed for potential clinical applications. Bortezomib (Velcade^®^), a nonselective proteasome inhibitor, has been widely used in the treatment of multiple myeloma [76]. However, due to its lack of specificity, bortezomib inhibits not only the proteasome but also several off-target proteins. This broad activity contributes to a high incidence of adverse effects, particularly peripheral neuropathy, which affects more than 30% of treated patients [77].

A major breakthrough in the field occurred in 2009 with the discovery of ONX 0914 (formerly PR-957), a selective inhibitor of the IMP catalytic subunit LMP7 (β5i) [78] whose selectivity can be explained by the difference between the S1 pockets of β5 and β5i [79]. The study, which reported the efficacy of ONX 0914 in mouse models of autoimmune diseases, represented a paradigm shift and caused widespread interest in the field. For the first time, it provided proof of concept that selective IMP inhibition could attenuate inflammation without broadly suppressing proteasomal function. While primarily targeting LMP7, ONX 0914 also inhibits LMP2 and MECL-1 subunits, though to a lesser extent [80]. Importantly, ONX 0914 exhibits at least 14-fold greater selectivity for LMP7 over β5c and does not significantly impair overall proteasome function.

In animal models, it effectively inhibited LMP7-dependent antigen presentation and suppressed the release of key pro-inflammatory cytokines, including IL-23, IFNγ, and IL-2, thereby demonstrating strong therapeutic potential [78]. Notably, ONX 0914 showed reduced systemic toxicity compared to nonselective proteasome inhibitors, underscoring its promise as a safer and more targeted therapeutic strategy for conditions associated with elevated IMP expression, such as chronic inflammatory diseases, neurodegenerative disorders, and certain cancers.

These seminal findings not only validated IMP as a druggable target but also sparked the development of additional IMP-specific compounds, such as KZR-616, a selective inhibitor of LMP2 and LMP7, which is 18- and 81-fold more selective for LMP7 and LMP2 than for their standard counterparts β5c and β1c, respectively, and is currently under clinical investigation for autoimmune and inflammatory conditions [81]. Another noteworthy inhibitor, M3258, is an orally bioavailable, potent, reversible, and highly selective LMP7 inhibitor with 305-fold greater selectivity for LMP7 over β5c. It has demonstrated a favorable safety profile in animal studies, with no observed adverse effects in vital organs, including the heart, lungs, kidneys, nervous system, and gastrointestinal tract [82]. In models of heart transplantation, DPLG3 has shown efficacy and demonstrated greater selectivity than ONX 0914 [83]. IPSI-001 has emerged as a more promising inhibitor that does not bind to β2c or β5c and exhibits a 100-fold preference for IMPs with low cytotoxicity, making it a valuable candidate for further investigation [84]. The development of selective LMP2 inhibitors presents greater challenges due to the high structural similarity among trypsin-like active sites [79]. Despite this, LU-002i was engineered to enhance selectivity, achieving a 45-fold preference for LMP2 [85]. Interestingly, rapamycin, a macrolide antibiotic, can also selectively inhibit IMPs without affecting the standard proteasome or ubiquitination. Its inhibitory action is partially attributed to suppression of NF-κB (nuclear factor kappa B), pathway activation, and reduced induction of IMPs by inflammatory stimuli [86]. A similar effect has been observed with the natural polyphenol resveratrol [87]. Although new IMP-selective inhibitors continue to be developed, most of these compounds are currently being tested in models of cancer, neurodegenerative, or autoimmune diseases [23,38,73,88,89,90,91].

## 3. Emerging Insights into the Role of Immunoproteasomes in Chronic Inflammation

IMPs were initially characterized by their enhanced ability to generate antigenic peptides for MHC-I presentation [92]. However, accumulating evidence indicates that their roles extend beyond adaptive immunity, revealing a central function in modulating innate immune responses, regulating cytokine production, and shaping immune cell phenotypes in the setting of chronic inflammation. In models of autoimmune and inflammatory diseases, such as rheumatoid arthritis (RA), systemic lupus erythematosus, inflammatory bowel disease (IBD), and multiple sclerosis, IMPs are markedly upregulated and have been shown to promote inflammation through activation of NLRP3 inflammasomes [93,94], amplifying the production of pro-inflammatory cytokines (e.g., IL-6, IL-17, TNFα), supporting the differentiation of pathogenic Th1 and Th17 cells, and sustaining pro-inflammatory M1 macrophage polarization [35,61,78]. Together, these findings highlight the pro-inflammatory functions of IMPs in chronic inflammation-mediated disorders.

Mechanistically, IMPs regulate the NF-κB signaling pathway, a central regulator of inflammatory responses, immune activation, and cellular survival [95], by promoting the degradation of the IκBα inhibitor, thereby enabling nuclear translocation of NF-κB and the transcription of inflammatory genes [55]. Mice deficient in IMPs display attenuated NF-κB signaling and reduced cytokine production and are protected against autoimmune disease in multiple preclinical models [96]. However, other lines of evidence challenge the view that IMPs are essential for NF-κB activation. For instance, in many cell types, standard proteasomes are sufficient to mediate IκBα degradation and activate NF-κB, even under inflammatory conditions [97]. Moreover, mice deficient in LMP7 or LMP2 retain residual proteasomal activity and often exhibit near-normal NF-κB signaling, suggesting that the constitutive proteasome can functionally compensate for the absence of IMP catalytic subunits [21]. Therefore, while the standard proteasome is clearly indispensable for NF-κB activation, the role of IMPs seems to be more nuanced and context- and cell-type-specific, which is particularly relevant under conditions of chronic inflammation. Rather than acting as primary drivers of NF-κB signaling, IMPs likely function as modulators that influence the magnitude, persistence, and immune consequences of the response.

IMPs are also implicated in the failure to resolve the age-associated, low-grade chronic inflammation that underlies multiple age-related diseases [22,98,99]. Persistent IMP expression in aging tissues may contribute to sustained immune activation and impaired tissue regeneration [100]. In this context, IMPs serve not only as biomarkers of chronic immune activation but also as potential therapeutic targets. As noted above, selective IMP inhibitors, such as ONX 0914, KZR-616, and others, have demonstrated potent anti-inflammatory and immunomodulatory effects in various inflammation models. These findings are summarized in Table 1.

Taken together, these data support a non-redundant, regulatory role for IMPs in the maintenance and amplification of chronic inflammation. Importantly, the development of selective IMP inhibitors has opened new therapeutic avenues. These compounds have shown promising results in preclinical models of chronic inflammatory disorders by dampening pathogenic immune responses while preserving immune surveillance.

## 4. Immunoproteasomes and Skeletal Muscle Pathologies: A Connection Gaining Recognition

Sarcopenia is an age-associated loss of skeletal muscle mass, strength, and function accompanied by severe adverse health outcomes, such as falls and fractures, functional decline, high health costs, and mortality [2,3]. Despite a considerable body of research, the etiology of sarcopenia remains unclear, and its pathogenesis is still poorly understood. Nevertheless, several major biological processes have been proposed to explain its pathogenesis. These include imbalances in muscle protein turnover [12], neuromuscular junction (NMJ) impairment [112], disturbed mitochondria biogenesis and function [113], insulin resistance [114], fat deposition in muscle [5,115], disturbed myokine production [116], impaired autophagy [117], enhanced muscle cell senescence [118], and non-resolved systemic, chronic, low-grade inflammation [6,119]. Notably, this inflammation has been proposed to link the pathogenesis of sarcopenia with obesity [5,120], type 2 diabetes mellitus [121], and metabolic-dysfunction-associated fatty liver disease (MAFLD) [122], thereby exacerbating sarcopenia manifestations. Altogether, these pathological events result in abnormal muscle fiber reorganization, myofibril degeneration, and myocyte death, as detailed in a series of reviews (e.g., [6,123,124,125,126,127]).

Although extensive research has been undertaken, no pharmacological therapy has yet proven effective for sarcopenia [128,129,130]. At present, the most beneficial strategies for mitigating its progression remain structured exercise programs, particularly resistance training, combined with targeted dietary interventions and supplementation [131]. In elderly individuals, who are often sedentary due to frailty or disability, nutritional management is considered the primary therapeutic option [132]. Among dietary factors, high-quality proteins and the amino acid leucine have shown the greatest promise in supporting skeletal muscle health. Importantly, aging is characterized by reduced muscle sensitivity to the anabolic effects of dietary intake, leading to impaired stimulation of muscle protein synthesis compared with younger adults [133,134,135].

Despite extensive studies in which the role of the UPS in protein degradation in sarcopenia has been repeatedly studied, contradictory findings have emerged, demonstrating, for example, both elevated and unchanged levels of atrogin-1 and MuRF1, two main E3 ubiquitin ligases that play a crucial role in muscle protein breakdown and are key players in muscle atrophy [136,137,138]. Nevertheless, accumulating evidence suggests direct and indirect roles of IMPs in the development of sarcopenia, in close association with chronic inflammation parameters. First, it has been demonstrated that skeletal muscle expresses IMPs; muscle biopsies from older adults revealed an upregulation of IMP subunits, particularly LMP7, which correlates with elevated IL-6 and TNFα expression [24]. Missense mutations in the IMP subunit LMP7 have been reported as the main pathogenic factors in Nakajo–Nishimura syndrome, a rare, inherited autoinflammatory disorder characterized by skeletal muscle atrophy [139,140]. Importantly, this syndrome is also associated with lymphocytic infiltration in the muscles and myositis, thus linking it to inflammation [130]. In a murine model of muscular dystrophy (BlAJ mice) caused by mutations in the dysferlin gene, which leads to muscle weakness and wasting and thus resembles sarcopenia, application of ONX 0914 ameliorated muscle pathology. This improvement was associated with anti-inflammatory M2 macrophage polarization, reduced muscle inflammation, and macrophage-mediated vessel stability, resulting in improved muscle performance in BlAJ mice [27]. In a murine denervation model of sarcopenia, increased LMP7 and MECL-1 expression in skeletal muscle has been found [141], which is suggested not to be directly responsible for muscle wasting but rather to trigger signaling events that ultimately enhance the proteolytic pathways of the cell. In LMP7/MECL-1 double KO (L7M1) mice, no major biochemical, histological, or functional differences were observed in skeletal muscle compared to wild-type controls [142]. However, following exercise, WT muscles upregulated LMP7, while L7M1 muscles did not, suggesting that IMPs may contribute to maintaining skeletal muscle homeostasis.

Recent data suggest a link between skeletal muscle senescence-associated deterioration, chronic inflammation, and IMPs. In *mdx* mice, a mouse model of human Duchenne muscular dystrophy (DMD), the accumulation of muscle-infiltrating macrophages expressing the senescence-associated secretory phenotype (SASP) release pattern, as well as senescence-related markers (such as p16 and β-gal), has been observed in association with the exhaustion of the skeletal muscle stem cell (satellite cell) pool in dystrophic muscle [143]. Potential involvement of IMPs in muscle cell senescence has been proposed based on studies of 9-month-old *mdx* mice, in which ONX 0914 administration resulted in an amelioration of the pathological features of muscle atrophy progression. This was associated with a reduced number of macrophages and effector memory T cells in both muscle and the spleen while increasing the number of Tregs and attenuating oxidative stress through improved mitochondrial efficiency [144,145]. Moreover, ONX 0914 mitigates oxidative stress by enhancing mitochondrial efficiency, which in turn leads to a significant reduction in fibrosis, restoration of muscle mechanical function, and improvement in muscle force [26]. Furthermore, these data suggest that the IMP-selective inhibitor ONX 0914 improves the already established and advanced pathological phenotype of aged *dystrophic muscle* by modulating multiple sarcopenia-associated pathways.

Lipotoxicity, induced by the accumulation of toxic lipid intermediates, such as diacylglycerols, ceramides, and free fatty acids, in skeletal muscle [146,147], is one of the key pathogenic factors in sarcopenic obesity, an age-associated syndrome in which the development of sarcopenia is exacerbated by coexisting obesity [5,148]. Ectopic fatty acids trigger oxidative stress, impair mitochondrial function, enhance insulin resistance [149], and induce production by skeletal muscle myokines, such as myostatin, CCL2, TNFα, IL-1, and IL-6 [150], thus promoting and supporting chronic inflammation [5]. Regarding IMPs, in a study of mice fed a high-fat diet (HFD) and subjected to acute eccentric exercise–induced muscle damage, an increase in skeletal muscle LMP7 and MECL-1 content was observed, along with reduced oxidative stress [151]. These findings suggest that exercise may protect obese individuals from muscle lipotoxicity through IMP upregulation. In another study of HFD-fed mice, although pro-inflammatory cytokines remained unchanged, oxidative protein damage was elevated in the gastrocnemius and tibialis anterior muscles. This intramuscular protein damage coincided with reduced IMP and total proteasome activity, as well as reductions in relative muscle mass, suggesting that proteasome dysregulation may be a critical link between obesity-related oxidative stress and muscle pathology [152]. Notably, LMP7-deficient HFD mice were resistant to obesity and displayed improved glucose tolerance and insulin sensitivity, accompanied by reduced inflammatory responses, such as macrophage infiltration and chemokine expression [153]. Furthermore, in skeletal muscle tissue from marmosets, inhibition of mTOR was associated with increased expression of PSMB5, a core subunit of the 20S proteasome, but not PSMB8, along with elevated expression of mitochondria-targeted protein chaperones [154]. Together, these findings suggest that alterations in IMP activity intersect with oxidative stress, inflammatory responses, and mitochondrial adaptations, thereby providing a mechanistic link between sarcopenia and IMP function.

In addition to sarcopenia, idiopathic inflammatory myopathies (IIMs), a group of complex disorders, are also characterized by significant loss of muscle mass and strength, accompanied by immune cell infiltration into muscle tissue and upregulation of MHC class I expression on myofibers [28]. For example, increased mRNA and protein expression of IMP subunits (PSMB8, PSMB9, and PSMB10) has been detected in muscle biopsies and peripheral blood cells of patients with polymyositis, dermatomyositis, and overlap-syndromes with myositis compared to those with non-inflammatory myopathies and healthy donors [29]. This upregulation correlated with elevated expression of T-cell-specific transcripts in active IIM muscles and was accompanied by increased expression of dendritic cell and monocyte marker genes, suggesting the therapeutic potential of IMP inhibitors for IIMs. Indeed, in a mouse model of polymyositis, administration of ONX 0914 or KZR-616 prevented loss of grip strength and reduced leukocyte infiltration into muscle tissue. Moreover, LMP7-deficient mice were resistant to the induction of polymyositis [30].

In a study of inflamed skeletal muscle tissues from patients with sporadic inclusion body myositis, immune-mediated necrotizing myopathies, and dermatomyositis, two IMP subunits, LMP2 and LMP7, were found to co-localize with MHC-class-I-expressing myofibers [155]. In addition, both myofibers and muscle-infiltrating cells, including CD8^+^ T cells and CD68^+^ macrophages, expressed LMP2 and LMP7. Selective inhibition or depletion of LMP7 enhanced TNFα- or IFNγ-mediated expression of cytokines/chemokines (myokines) in myoblasts. Furthermore, specific inhibition of LMP2 or LMP7 reduced IFNγ-induced MHC class I surface expression in myoblasts.

Elevated LMP7 expression was also detected in the muscles of patients with dermatomyositis [156]. In that study, LMP7 overexpression in human skeletal muscle myoblasts (HSMMs) significantly upregulated the muscle atrophy marker MuRF1, type I IFN-related proteins (MxA and IFNβ), and NF-κB pathway-related proteins (pIκBα, pIRF3, and pNF-κBp65). Moreover, LMP7 overexpression markedly reduced HSMM viability, which was partially restored through treatment with the LMP7 inhibitor PR957. In patients with myositis associated with anti-Ku autoantibodies, a rare inflammatory myopathy characterized by prominent upregulation of autophagy, myofiber necrosis, MHC class I and II positivity, and variable endomysial inflammation, conspicuous sarcoplasmic protein aggregates were found to be LMP7-positive [157]. In a murine model of autophagy deficiency, inhibition of eukaryotic translation initiation factor 4E (EIF4E) improved 20S proteasome activity in skeletal muscle, an effect attributed to increased expression of IMP subunits LMP2 and MECL [158]. These findings suggest a regulatory role for IMPs in autophagy within skeletal muscle.

## 5. Inflammasome Inhibitors in Skeletal Muscle Pathologies: A Double-Edged Sword

Findings discussed in the section above suggest that while the standard proteasome is responsible for degrading myofibrillar proteins during muscle atrophy [137], IMPs may play a role in regulating the inflammatory crosstalk between muscle cells and infiltrating immune cells. Maladaptive immune activation in skeletal muscle pathologies may therefore be exacerbated by IMP-mediated cytokine production and antigen presentation, establishing a vicious cycle of inflammation and muscle wasting [5]. However, IMPs may also protect aged muscle by maintaining proteostasis and supporting myogenesis, particularly during regeneration [25]. Thus, the impact of IMPs in skeletal muscle pathologies appears to be context dependent; they may be protective when facilitating muscle differentiation and protein quality control but detrimental when driving chronic inflammation. This dual role makes them a promising, yet potentially risky, therapeutic target in skeletal muscle pathologies, depending on the timing, disease stage, and balance between proteostatic maintenance and immune activation. This duality raises an important, yet unresolved, question about the rationale for using IMP inhibitors in skeletal muscle pathologies. In other words, “To use or not to use IMP inhibitors in skeletal muscle pathologies—that is the question”.

Why might IMP inhibitors be beneficial? (i) Selective IMP inhibitors (e.g., targeting LMP7 or LMP2) do not shut down all proteasome activity. The constitutive proteasome remains functional, allowing for baseline protein degradation [90,159]. (ii) IMP inhibitors suppress overactive immune signaling without fully blocking proteostasis—reducing cytokine production, antigen presentation, and immune cell infiltration [23]. (iii) IMP inhibition decreases Th1 and Th17 cell differentiation—key drivers of chronic inflammation [62]), limits activation of autoreactive CD8^+^ T cells [22], and dampens excessive macrophage and dendritic cell cytokine production [160]. (iv) This selective immune modulation can reduce muscle inflammation, as demonstrated in models of IIMs [30], DMD [134], and even systemic inflammation [75].

What might be the detrimental consequences of the application of IMP inhibitors in skeletal muscle pathologies? (i) Inhibiting IMP activity can impair the clearance of damaged proteins, increase proteotoxic stress, and disrupt mitochondrial function, especially in aged or metabolically compromised muscle [72,73,161,162]. (ii) Inhibiting IMP activity can impair the regulation of muscle–immune cell crosstalk, helping to balance pro- and anti-inflammatory signals, leading to suppression of essential immune surveillance, and reduce satellite cell activation, ultimately hindering muscle repair [26,145,163].

Thus, while selective IMP inhibitors may offer benefits by dampening chronic inflammation, their impact on muscle regeneration and tissue homeostasis is context-dependent. In skeletal muscle pathologies, where muscle plasticity is already compromised, untargeted or poorly timed IMP inhibition may worsen outcomes. This underscores the importance of careful therapeutic timing, individualized patient stratification, and a clear understanding of disease stage when considering IMP-based interventions.

To overcome the potential negative effects of IMP inhibitors, combination therapy may offer a promising solution. This approach has been validated in several preclinical models of autoimmune disease [164]. Specifically, the combined inhibition of LMP7 and LMP2, using the selective LMP7 inhibitor PRN1126 alongside LMP2 inhibitors, such as LU-001i or ML604440, was shown to reduce MHC-I surface expression on splenocytes, suppress IL-6 secretion, impair Th17 differentiation, and alleviate symptoms in models of experimental colitis and experimental autoimmune encephalomyelitis (EAE) [163]. Moreover, in a mouse model of anti-collagen antibody-induced arthritis, monotherapy with selective single-site inhibitors had limited efficacy, while the combination of LMP7 with MECL-1 or LMP2 inhibitors produced a clear therapeutic benefit. Notably, the combination of LMP2/MECL-1 with LMP7 inhibition is also effective in constitutive proteasome-resistance cells [165]. Collectively, these findings suggest that effective modulation of IMP activity in inflammatory conditions may require the simultaneous targeting of multiple catalytic subunits. Although data on IMP inhibitor combinations in skeletal muscle-associated disorders are currently lacking, the central role of chronic inflammation in the pathogenesis of skeletal muscle pathologies supports the rationale for exploring such combinatorial strategies in skeletal muscle pathologies.

## 6. Concluding Remarks

Skeletal muscle pathologies are characterized by progressive muscle wasting that is strongly influenced by age-related chronic inflammation. Within this context, IMPs have emerged as intriguing but paradoxical regulators of muscle homeostasis. Mounting evidence suggests that IMPs may exert both protective and detrimental effects depending on the biological context, disease stage, and cellular environment. On the one hand, IMPs support protein homeostasis and contribute to myogenic differentiation, especially under conditions of cellular stress or regeneration. Their role in maintaining antigen processing and modulating cytokine production may also serve beneficial immune surveillance functions in aging tissue. On the other hand, persistent IMP activation, common in chronic inflammatory states, can exacerbate muscle degradation by amplifying immune responses, sustaining cytokine production, and facilitating maladaptive interactions between muscle cells and infiltrating immune populations. This duality underpins the current uncertainty surrounding the therapeutic use of IMP inhibitors in skeletal muscle pathologies.

Preclinical studies using selective IMP inhibitors (e.g., ONX 0914, KZR-616) in models of autoimmune myositis or neurogenic atrophy have shown promising anti-inflammatory effects and muscle-sparing properties. However, these findings are not easily translatable to skeletal muscle pathologies, where muscle plasticity is already diminished and regenerative capacity is impaired. Inhibiting IMPs in this setting might further compromise proteostasis, impair satellite cell activation, and disrupt immune–satellite cell crosstalk essential for muscle repair. Moreover, unresolved questions remain about the timing, specificity, and combinatorial targeting of IMP subunits. Some studies suggest that selective dual inhibition (e.g., LMP7 and LMP2) yields superior therapeutic efficacy in inflammatory models compared to single-subunit inhibition. Whether such approaches can be safely and effectively applied to skeletal muscle pathologies is unknown and warrants focused investigation.

In summary, the role of IMPs in skeletal muscle pathologies is complex and context-dependent. Their inhibition holds therapeutic promise but may also pose risks to muscle integrity and regeneration. A deeper understanding of IMP function across different stages of muscle aging and inflammation is urgently needed to develop rational, targeted interventions. Future studies should prioritize patient stratification, biomarker development, and integrative approaches that balance immune modulation with preservation of muscle health.

**Figure 2:**
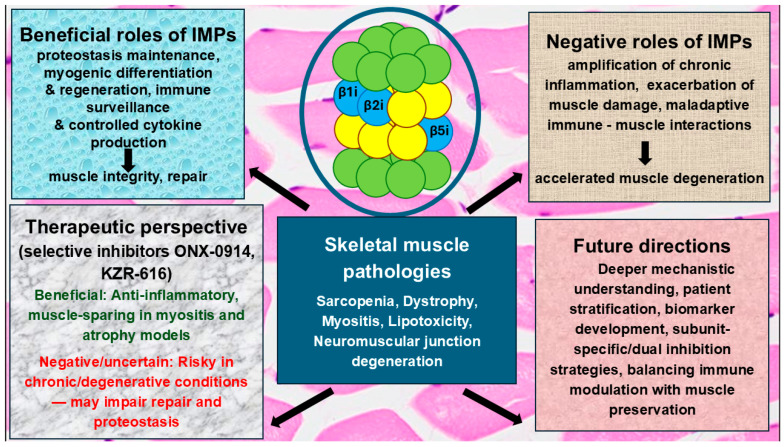
Schematic representation of the roles of immunoproteasomes (IMPs) in skeletal muscle pathologies. IMPs play dual roles in skeletal muscle pathologies, supporting repair and homeostasis on one side but driving inflammation and degeneration on the other, with therapeutic strategies aiming to balance these opposing effects.

In summary, the role of IMPs in skeletal muscle pathologies is complex and context-dependent. Their inhibition holds therapeutic promise but may also pose risks to muscle integrity and regeneration. As illustrated in Figure 2, a deeper understanding of IMP function across different stages of muscle aging and inflammation will be essential to develop rational, targeted interventions. Future studies should prioritize patient stratification, biomarker development, and integrative approaches that balance immune modulation with the preservation of muscle health.

## Figures and Tables

**Table 1:** Effects of immunoproteasome inhibitors in inflammation models.

Name and Target	Selectivity	Model	Effects and Mechanisms	Refs.
ONX-0914, LMP2, and LMP7	LMP7 subunit (20- to 40-fold more selective than β5c or LMP2)	Murine PBMC culture; murine collagen-induced arthritis (CIA)	Reduces the production of IL-23 by activated monocytes and IFNγ and IL-2 by T cells. Attenuates the severity of arthritis, reduces cellular infiltration, IL-1β, IL-6, and TNFα production, and autoantibody levels.	[78]
ONX-0914		Murine lupus model	Prevents nephritis progression, decreases serum autoantibody levels, and reduces IFNα production of TLR-activated plasmacytoid dendritic cells.	[101]
ONX-0914		Murine models of psoriasis, spontaneously developing and imiquimod-induced	Reduces skin thickness, inflammation scores, and pathological lesions, normalizes the expression of several pro-inflammatory genes in the ear and significantly reduced the inflammatory infiltrate, accompanied by a significant alteration in the αβ^+^ and γδ^+^ T cell subsets.	[102]
ONX-0914		Murine models of atherosclerosis	Reduces atherosclerosis, dendritic cell, and macrophage levels and their activation and the levels of antigen-experienced T cells and Th1 cells. Reduces accumulation of neutrophils and macrophages in white adipose tissue, intestinal triglyceride uptake and gastric emptying, and improves markers of metabolic syndrome.	[103]
ONX-0914		Human skeletal muscle myoblasts	Recovers reduced cell viability after LMP7 overexpression.	[104]
ONX-0914		PBMCs of immune thrombocytopenia (ITP) patients; ITP murine model	Increases the number of platelets, decreases the expression of FcγRI in ITP mice and decreases that of FcγRIII in ITP patients, inhibits the activation of CD4^+^ T cells, and affects the differentiation of Th1 cells in patients with ITP.	[105]
ONX-0914	LU-102, inhibitor of β2 (MECL-1)	Human LN229, GBM8401, and U87MG glioblastoma cells; orthotopic mouse glioblastoma model	Induces cell cycle arrest, apoptosis, and autophagy; reduced BCL-2 expression of ONX-0914 also induced in glioblastoma cells. In vivo, TMZ plus ONX-0914 reduced tumor progression better than the control or TMZ alone.	[106]
ONX-0914		Castration-resistant prostate cancer (CRPC) tumor graft model	Suppresses the “tumor-elicited” Th17-type inflammatory response, angiogenesis, and epithelial–mesenchymal transition via inactivation of COX-2/VEGF-A signaling and β-catenin/Snail signaling.	[107]
ONX-0914 and LU-102		Human multiple myeloma (MM) cell lines	ONX-914 induces MM cell cytotoxicity, which is enhanced by IFNγ. LU-102, and dramatically sensitizes MM cells ONX-0914. ONX-0914 synergizes with all FDA-approved proteasome inhibitors in MM in vitro and in vivo.	[108]
YU102 and LMP7		Murine model of LPS-induced neuroinflammation	Attenuates disease progression, reduces the number of reactive astrocytes and microglia, and suppresses the secretion of IL-1α, IL-1β, and CCL12 from microglial cells.	[109]
KZR-616, LMP2, and LMP7	LMP7 and LMP2 subunits (18- and 81-fold more selective than β5c and β1c)	Murine lupus model, healthy volunteers	Completely resolves proteinuria mediated by alterations in T and B cell activation, including reduced numbers of short- and long-lived plasma cells in mice. Selectively inhibits immunoproteasomes and blocks cytokine production following ex vivo stimulation.	[81]
KZR-616		Murine model of C-protein-induced myositis	Similarly to ONX 0914, it prevents loss of grip strength, reduces leukocyte infiltration of the muscle, and prevents increased serum creatine kinase levels.	[30]
PKS3053 and LMP7	LMP7 subunit (5600- and 13,600-fold more selective than β5c)	Human PBMCs, murine model of cutaneous lupus erythematosus	Reduces TLR-dependent activation of plasmacytoid dendritic cells and decreases their maturation, IFNα response, and T cell proliferation. Decreases inflammation, cellular infiltration, and skin damage.	[110]
M3258 and LMP7	LMP7 subunit (>500-fold more selective than β5c)	Human breast cancer (BC) samples and cell lines, murine BC model	Reduces viability and induced cell apoptosis in vitro, reduces tumor growth and the tumor abundance of M2 macrophages, activates tumor-infiltrating CD8+ T cells, and suppresses the expression of specific inflammatory pathway gene signatures in immune cells.	[111]
DPLG3 and LMP7	LMP7 subunit (7200-fold more selective than β5c)	Murine dextran sulphate (DSS)-induced colitis	Attenuates disease progression and decreases the production of IL-6, IL-1β, IFNγ, and TNFα and the influx of effector T cells and macrophages in colon tissues while increasing the number of Tregs; reduces the expression of NF-κB p50 and p65.	[112]

Abbreviations: CCL12, chemokine (C-C motif) ligand 12; PBMC, peripheral blood mononuclear cells; TLR, toll-like receptor; Tregs, T-regulatory cells.

## Data Availability

No data were used for the research described in the article.

## Abbreviations

CCL2, CC motif chemokine ligand 2; CVD, cardiovascular disease; DMD, Duchenne muscular dystrophy; EAE, experimental autoimmune encephalomyelitis; IBD, inflammatory bowel disease; IFN, interferon; INF-κB, nuclear factor kappa B; IIMs, idiopathic inflammatory myopathies; IL, interleukin; IMP, immunoproteasome; HFD, high-fat diet; LMP2, low molecular mass polypeptide 2; MECL1, multicatalytic endo-peptidase complex–like; MHC-I, major histocompatibility complex class; MuRF1, muscle RING-finger protein 1; MxA, myxovirus resistance protein A; NMJ, neuromuscular junction; PBMC, peripheral blood mononuclear cells; pIκBα, phosphorylated inhibitor of kappa light polypeptide gene enhancer in B-cells, alpha pIRF3, phosphorylated interferon regulatory factor 3; POMP, proteasome maturation protein; PSMB9, proteasome subunit beta type-9; RA, rheumatoid arthritis; SASP, senescence-associated secretory phenotype; TLR, toll-like receptor; Th, T helper cell; Treg, regulatory T cell; UPS, ubiquitin–proteasome system.
